# 

**Published:** 1858-01

**Authors:** 


### INDEX TO VOL. V.

OF

### HALL'S JOURNAL OF HEALTH.

PAGE

A Pure Joy.................. 16
Antidote for Yellow Fever........ 48
Aids to Digestion.............63, 232
Air and Exercise........71, 152, 255
Alum in Bread.................. 90
Abernethy Cured of Dyspepsia... 130
Bible Health-teachings..........5, 97
Be Thankful...................... 21
Best Student..................... 27
Blacking Boots................... 28
Breakfast, Early................. 33
Best Preachers................... 36
Bathing.......................... 40
Bread...............42, 90, 214
Blessed Charity..............75, 272
Borden's Condensed Milk......... 107
Benton, Thomas H., Death of.... 121
Bites, Poisonous, Cured.......... 134
Boils, Cure of..............135, 230
Balm of Gilead.................. 144
Baldness........................ 274
Cure-All........................ 265
Children, Training......13, 19, 37, 109
Clerical Exposures, 15, 117, 176, 183, 245
Consumption.............23, 185, 248
Constipation Avoided............ 183
Condensed Milk.................. 107
Chat about Health..........115, 182
Cancer.......................... 121
Coffee and Tea......131, 167, 237, 257
Chloroform Hallucination......... 255
Chapped Hands.................. 290
Disease and Poverty............. 263
Dyspepsia........32, 63, 123–5, 130–2
Drunkenness Cured............... 156
Danger to Orators........61, 133, 140
Doctors........109, 119, 143, 230, 233

PAGE

Dangerous Receipts.............. 289
Employments, Health of.......26, 191
Eating...17, 33, 73, 124, 128, 192, 277
Exercise and Air.........71, 124, 256
Edwards, Jonathan, Eating Trials. 128
Epilepsy......................... 173
Early Rising..................... 203
Eggs (to know good)............. 273
Fever and Ague Prevented.....30, 217
Food of Infants.................. 99
Fruits, Use of.......94, 123, 141, 202
Forethought...................... 197
Feathers and Fog................. 260
Fame............................. 261
Food Cure........................ 274
First Step Awry.................. 279
Gratuitous Medical Advice.....25, 143
Greatness and Health............. 38
Gluttony......................... 89
Going Down....................... 117
Grumbling at Meals............... 258
Health of Employments.......26, 191
Heart Affections................. 162
Health a Duty........39, 49, 115, 235
Hair Specifics................... 179
Health of Cities..............72, 97
Horseback Exercise............... 113
Hard Study Healthful............. 164
How to Get Sick.................. 138
Houses Warmed.................... 266
Hard to Take..................... 277
How to Rejuvenate................ 288
"Health and Disease"............. 291
Insanity......................... 59
Inhalation, Medicated............ 72
Infants' Food.................... 99
Insect Bites Cured............... 134

PAGE
Inverted Toe-nails, ... 239
"Journal's" True Estimate ... 290
Kindness Rewarded ... 282
Liquor and Tobacco ... 18, 88, 91, 133
Life-Long Lunatic ... 24
Late Suppers ... 31, 252
Longevity of Slaves ... 43
Loss of Voice ... 74
Liver and Stomach ... 135
Leaning on Providence ... 144
Marriage ... 11
Miscellanies ... 17, 145
Monumental Heights ... 28
Malarial Precautions ... 30
Ministerial Support ... 35
Medicated Inhalation ... 72
Milk, Bad ... 99, 105
Moths Exterminated ... 114
Make Yourself Useful ... 281
Novel-Reading Pernicious ... 46, 112
New York Mortuary ... 72
Nurses and Nursing ... 87
Nutrition and Stimulation ... 91
Night Air ... 216
Out-Door Safety ... 16
Orchard Planting ... 52
Oratory, Dangers of ... 15, 61, 117, 140
Over-eating ... 73
Onions, Nutritious ... 99
Our Troubles ... 110
One Calling ... 143
Pigs and Men ... 17, 133
Pulpit Power ... 34, 133
Passing Away ... 41
Parlor Ornaments ... 50
Planting Trees ... 52
Pills by the Bushel ... 95
Poor Man's Book ... 97
Poisonous Milk ... 105
Potato, New ... 134
Patent Medicine Swindles ... 134, 142
Providence Reposed in ... 144
Paste ... [illegible]
Reading, Fictitious ... 46
Riddance of Rats and Vermin, 49, 92, 144
Relapses ... 116
Recreations ... 121, 187, 211
Railroad Travelling ... 287
School Children ... 19, 154, 241
Suppers, Late ... 31, 139, 169
Safe Reading ... 46
Sea Voyages ... 64
Sabbath Hygiene ... 65, 188
Sneezing and Stupidities ... 86, 131, 190
Stimulation and Nutrition ... 91
Sleep, Delicious ... 108, 163, 238
Sprains ... 153
Saleratus and Soda Hurtful ... 124
Stitch in the Side ... 131
Stings of Insects Cured ... 134
Sewing-Machines ... 135
Stomach Managed ... 136, 252
Spectacles (use of) ... 263
Stammering ... 209
Sons and Daughters ... 269
Sight Injured ... 194
Things to Avoid ... 14, 33
Tobacco and Liquor ... 18, 133
Thanksgiving Dinner ... 20
Thankfulness, Cause for ... 21
Trouble ... 119, 160, 161
Travelling for Health ... 253
Throat-Ail ... 77, 132
Temperate Eating ... 136, 128, 228
Tom-Boys ... 283
Victoria as a Mother ... 41
Vinegar, the Best ... 63, 262
Voice, Lost ... 74
Vermin Riddance ... 49, 92, 140, 144
Warm and Cold Baths ... 40
Warts Cured ... 134
Wet Blankets ... 284
Wreck of All ... 286
Yellow Fever Antidote ... 48

